# VARS2 Depletion Leads to Activation of the Integrated Stress Response and Disruptions in Mitochondrial Fatty Acid Oxidation

**DOI:** 10.3390/ijms23137327

**Published:** 2022-06-30

**Authors:** Elham Kayvanpour, Michael Wisdom, Maximilian K. Lackner, Farbod Sedaghat-Hamedani, Jes-Niels Boeckel, Marion Müller, Rose Eghbalian, Jan Dudek, Shirin Doroudgar, Christoph Maack, Norbert Frey, Benjamin Meder

**Affiliations:** 1 Department of Cardiology, Angiology and Pneumology, University Hospital Heidelberg, 69120 Heidelberg, Germany; elham.kayvanpour@med.uni-heidelberg.de (E.K.); wisdom@stud.uni-heidelberg.de (M.W.); maximilian.lackner@med.uni-heidelberg.de (M.K.L.); farbod.sedaghat@med.uni-heidelberg.de (F.S.-H.); rose.eghbalian@med.uni-heidelberg.de (R.E.); norbert.frey@med.uni-heidelberg.de (N.F.); 2 DZHK (German Centre for Cardiovascular Research), Partner Site Heidelberg/Mannheim, 10785 Berlin, Germany; 3 Clinic and Polyclinic for Cardiology, University of Leipzig, 04109 Leipzig, Germany; jes-niels.boeckel@medizin.uni-leipzig.de; 4 Clinic for General and Interventional Cardiology/Angiology, Herz- und Diabeteszentrum NRW, University Hospital of the Ruhr-Universität Bochum, 32545 Bad Oeynhausen, Germany; mamueller@hdz-nrw.de; 5 Comprehensive Heart Failure Center (CHFC), University Hospital, Julius-Maximilians University of Würzburg, 97070 Würzburg, Germany; dudek_j@ukw.de (J.D.); maack_c@ukw.de (C.M.); 6 Department of Internal Medicine and the Translational Cardiovascular Research Center, University of Arizona—College of Medicine Phoenix, Phoenix, AZ 85004, USA; sdoroudgar@arizona.edu; 7 Klaus Tschira Institute for Computational Cardiology, 69120 Heidelberg, Germany; 8 Department of Medicine III, University of Heidelberg, INF 410, 69120 Heidelberg, Germany

**Keywords:** VARS2, heart failure, integrated stress response, mitochondrial FAO

## Abstract

Mutations in mitochondrial aminoacyl-tRNA synthetases (mtARSs) have been reported in patients with mitochondriopathies: most commonly encephalopathy, but also cardiomyopathy. Through a GWAS, we showed possible associations between mitochondrial valyl-tRNA synthetase (VARS2) dysregulations and non-ischemic cardiomyopathy. We aimed to investigate the possible consequences of VARS2 depletion in zebrafish and cultured HEK293A cells. Transient VARS2 loss-of-function was induced in zebrafish embryos using Morpholinos. The enzymatic activity of VARS2 was measured in VARS2-depleted cells via northern blot. Heterozygous VARS2 knockout was established in HEK293A cells using CRISPR/Cas9 technology. BN-PAGE and SDS-PAGE were used to investigate electron transport chain (ETC) complexes, and the oxygen consumption rate and extracellular acidification rate were measured using a Seahorse XFe96 Analyzer. The activation of the integrated stress response (ISR) and possible disruptions in mitochondrial fatty acid oxidation (FAO) were explored using RT-qPCR and western blot. Zebrafish embryos with transient VARS2 loss-of-function showed features of heart failure as well as indications of CNS and skeletal muscle involvements. The enzymatic activity of VARS2 was significantly reduced in VARS2-depleted cells. Heterozygous VARS2-knockout cells showed a rearrangement of ETC complexes in favor of complexes III_2_, III_2_ + IV, and supercomplexes without significant respiratory chain deficiencies. These cells also showed the enhanced activation of the ISR, as indicated by increased eIF-2α phosphorylation and a significant increase in the transcript levels of ATF4, ATF5, and DDIT3 (CHOP), as well as disruptions in FAO. The activation of the ISR and disruptions in mitochondrial FAO may underlie the adaptive changes in VARS2-depleted cells.

## 1. Introduction

Genome-wide association studies (GWASs) have been very successful in identifying novel variant–trait associations. In 2014, we showed through a large GWAS that the single-nucleotide polymorphism (SNP) rs9262636, located in a non-coding region of chromosome 6, is associated with dilated cardiomyopathy (DCM), described as the systolic dysfunction and dilatation of the left ventricle (LV) in the absence of coronary artery disease or abnormal loading conditions [1,2]. Further expression quantitative trait loci (eQTL) analysis in the blood of healthy volunteers found mitochondrial valyl-tRNA synthetase (VARS2) mRNA to be significantly upregulated in individuals carrying the minor allele G [1]. VARS2 is one of the nuclear-encoded mitochondrial aminoacyl-tRNA synthetases (mtARSs) and belongs to the class-I aminoacyl-tRNA synthetase family. It mediates the formation of carbon–oxygen bonds in aminoacyl-tRNA and facilitates the conjugation of the amino acid valine to its cognate tRNA molecule. It also possesses the ability to edit mischarged tRNAs, and its variations may lead to mistranslation [3]. Whereas variants in VARS2 have been associated with a better breast cancer prognosis and a higher risk of developing chronic hepatitis B [4,5], homozygous or compound heterozygous mutations in this gene have been found to cause mitochondriopathies, with encephalopathy being the most common, but cardiomyopathy and pulmonary hypertension also occurring [6,7,8,9,10,11,12,13,14]. In the present study, we investigated the consequences and thus the possible disease mechanisms of VARS2 depletion in two different model systems, zebrafish embryos and cultured HEK293A cells.

## 2. Results

VARS2 is expressed ubiquitously, including in tissues with a higher energy turnover, such as the cerebellar hemisphere, cerebellum, and left ventricle. Thirteen transcripts have been described in humans, of which six are protein-coding. Isoform 1 (ENST00000321897.9), with 29 coding exons, 4073 bps, and 1066 amino acids, is the most abundant protein-coding isoform in human left ventricles (data source: GTEx Analysis Release V8 (dbGaP Accession phs000424.v8.p2 on 03/03/2022)) (Figure S1).

### 2.1. Transient VARS2 Knockdown Leads to Heart Failure in Zebrafish 

**Figure 1:**
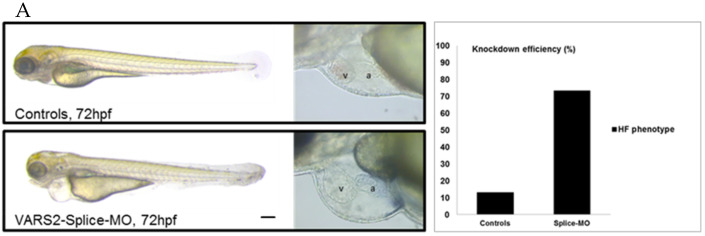

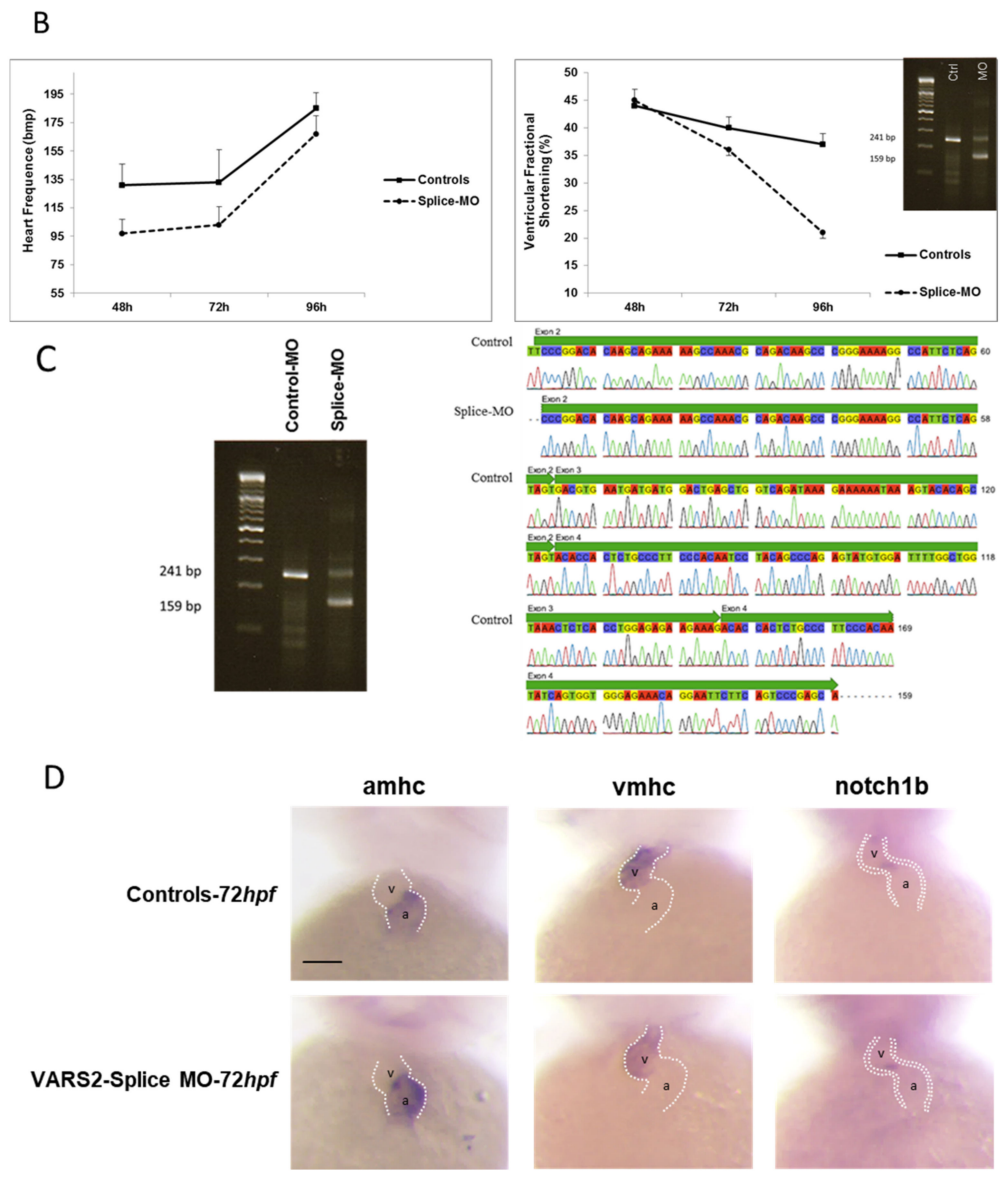
Phenotype of VARS2-MO-injected embryos in comparison to control-MO-injected embryos with 73% knockdown efficiency: *n* = 100 control-MO- and *n* = 100 splice-MO-injected embryos. Scale bar: 0.5mm (**A**). Heart rate and ventricular fractional shortening in control-MO- and splice-MO-injected embryos at 48h, 72h, and 96h post-fertilization (*n* = 25 each) (**B**). Gel electrophoresis showing a fraction of wild-type cDNA consisting of exons 2, 3, and 4 (241 bp) in control-MO-injected embryos and both wild-type and spliced cDNAs in splice-MO-injected embryos, with spliced cDNA missing exon 3 (159bp) (**C**). Whole-mount RNA antisense in situ hybridization showing expressions of atrial- and ventricle-specific myosin heavy chains as well as notch1b (**D**). v: ventricle, a: atrium, hpf: hours post-fertilization. Scale bar: 100µm.

We investigated the effect of transient VARS2 loss-of-function in zebrafish embryos using antisense oligonucleotides (Morpholinos). In comparison to control-MO-injected embryos, VARS2-MO-injected embryos showed the absence of exon 3 in the mature VARS2 mRNA and thus very early protein termination. The VARS2 morphants showed disturbed cardiac contractility (decreased fractional shortening) and bradycardia, as well as dilated ventricles, manifest pericardial edema, and pericardial blood congestion, all hallmarks of heart failure in zebrafish. Furthermore, some embryos showed cerebral edema and a curved back, suggesting CNS involvement and skeletal muscle affection. The knockdown efficiency was 73% (Figure 1A–C). Whole-mount RNA antisense in situ hybridization revealed the normal expression of atrial- and ventricle-specific myosin heavy chains as well as notch1b, suggesting normal molecular chamber and cardiac cushion specifications (Figure 1D).

### 2.2. VARS2-Depleted HEK293A Cells Showed Reduced Enzymatic Activity

**Figure 2:**
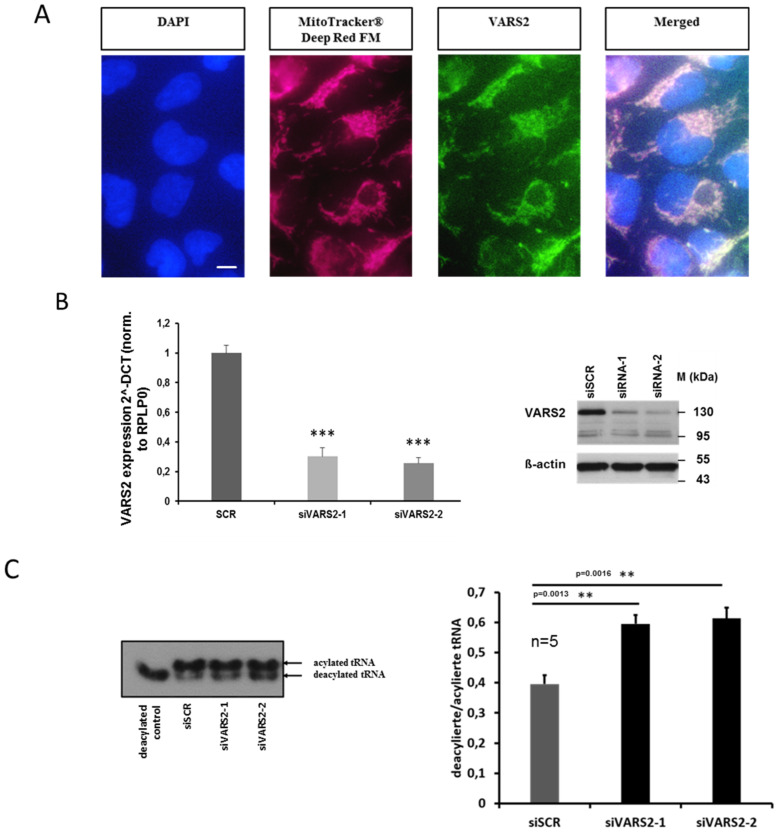
VARS2 is mainly localized in the mitochondria of HEK293A cells. The scale bar is 10 µm long (**A**). VARS2 expression is significantly reduced according to mRNA and protein levels in HEK293A cells treated with VARS2-specific siRNAs compared to control siRNA (**B**). HEK293A cells treated with VARS2-specific siRNAs showed significantly increased diacylation/acylation ratio in valyl-tRNA according to northern blot analysis. *n* = 5, siSCR mean = 0.4, SD = 0.07; siVARS2-1 mean = 0.59, SD = 0.07, *p* = 0.0013; siVARS2-2 mean = 0.61, SD = 0.08, *p* = 0.0016 (**C**). **≤0.01, ***≤0.001.

Using MitoTracker Deep Red FM as well as a VARS2-specific antibody, we first verified that VARS2 is indeed mainly localized in the mitochondria of HEK293A cells (Figure 2A). In order to evaluate the enzymatic activity of VARS2, after charging valyl-tRNA with amino acid valine, we implemented denaturing polyacrylamide gels, which separate charged tRNA from uncharged tRNA, followed by northern blot to determine the charged fraction of valyl-tRNA in the cultured cells. We found an increased deacylation/acylation ratio in the valyl-tRNA after treating the HEK293A cells with VARS2-specific siRNAs compared to the control siRNA, indicating decreased VARS2 activity (Figure 2B–C).

### 2.3. Heterozygous VARS2 Knockout (VARS2^+/−^ Knockout) was Successfully Achieved in HEK293A Cells

By implementing CRISPR/Cas9 technology, VARS2^+/−^ knockout was successfully established in HEK293A cells. As described in the Materials and Methods, this model was achieved by co-transfecting HEK293A cells with one plasmid encoding a CRISPR/Cas9 system targeting the VARS2 gene alongside another plasmid encoding a donor template with flanking homologous arms. Genomic PCR and sequencing, as well as RT-qPCR and western blot, confirmed the presence of one wild-type (WT) as well as one gene-edited allele, indicating that this cell line had a heterozygous knockout. RT-qPCR analyses showed visible knockout during and after the edit. Western blot analyses showed a reduced amount of VARS2 protein, whereas the total amount of mitochondrial protein as indicated by the VDAC2 content remained unchanged (Figure S2A–C). To our knowledge, this is the first report of an in vitro VARS2-knockout cell model created using CRISPR/Cas9 technology.

### 2.4. VARS2 Depletion Leads to Rearrangement of the Electron Transport Chain (ETC) Complexes without Significant Respiratory Chain Deficiencies

**Figure 3:**
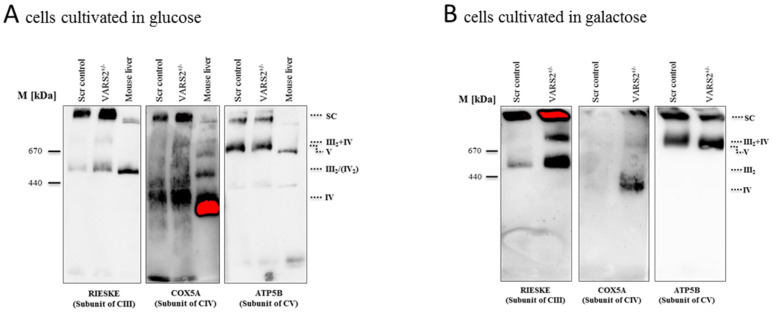

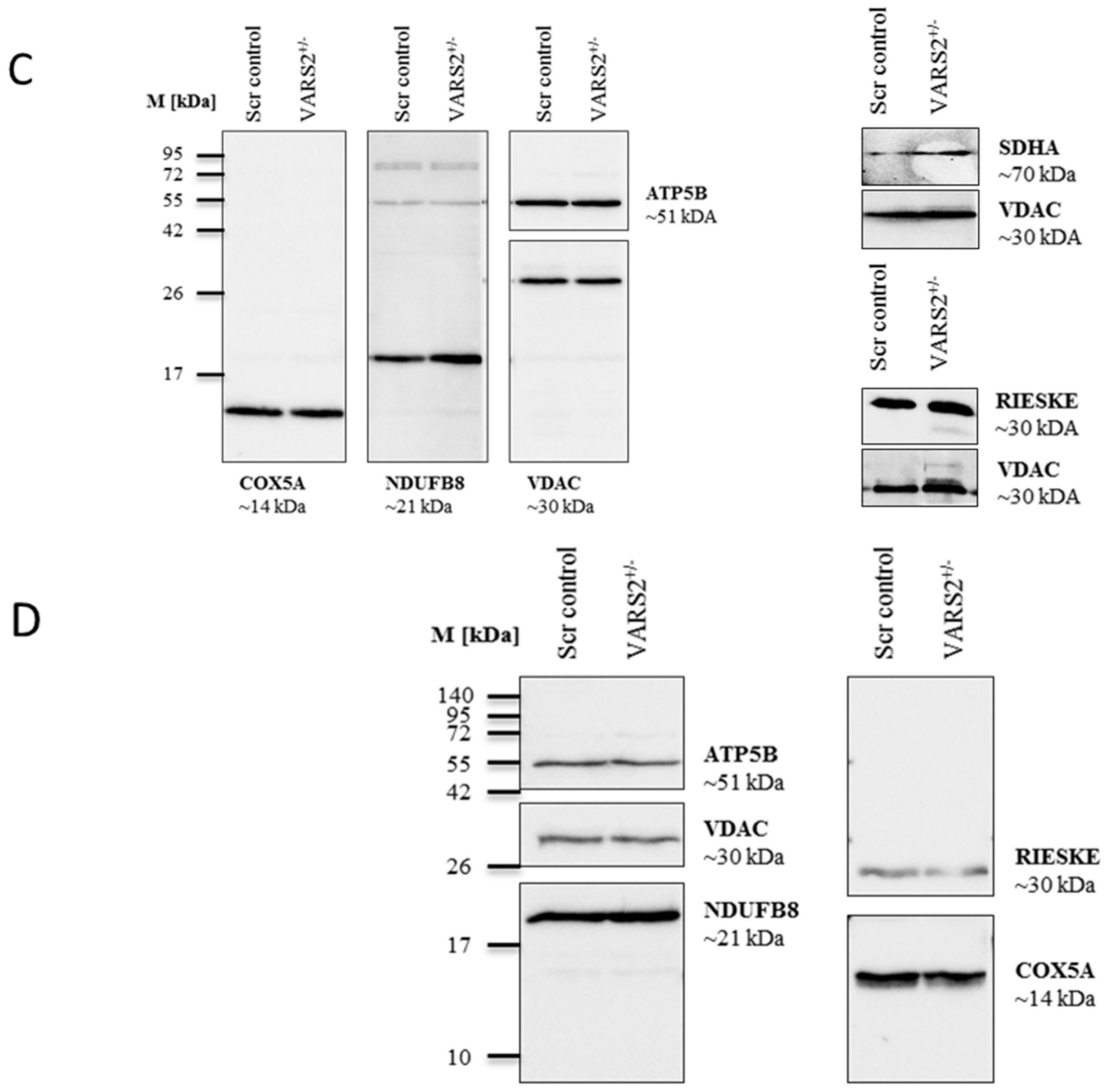
BN-PAGE analysis of respiratory chain supercomplex in mitochondria isolated from cells grown in glucose (**A**) or galactose (**B**). Western blot analysis of denatured respiratory subunits in mitochondria isolated from cells cultivated in glucose (**C**) or galactose (*n* = 1) (**D**). Mouse liver was run as a positive control.

To determine whether the decreased expression of VARS2 leads to changes in the electron transport chain (ETC) complex arrangement, we performed blue native polyacrylamide gel electrophoresis (BN-PAGE). This showed a marked increase in the levels of complex III dimers (III_2_), III_2_ + IV, and supercomplexes formed by two units of CIII and a variable number of CIV units in the presence or absence of one unit of CI (Figure 3A). This change was even more pronounced in cells that were forced to utilize mitochondrial respiration rather than glycolysis by cultivation in galactose (Figure 3B). However, the SDS-PAGE and the western blot analyses carried out showed no significant changes in the abundance of each respiratory subunit in the cells cultivated in glucose or galactose, not even in subunits of complex III or IV (Figure 3C–D). This may suggest that although the total expression of each respiratory subunit did not change, the complex arrangement changed following VARS2 depletion. In order to assess the energy metabolism in the VARS2 KO^+/−^ cells, high-resolution respiration measurements with a Seahorse XFe96 Analyzer were performed. Compared to the control cell line, the VARS2 KO^+/−^ cells showed no significant changes in the basal (*p* = 0.32), or maximal oxygen consumption rate (OCR) (*p* = 0.05) under standard cultivation conditions (Table S1 and an example run is shown in Figure S3A). The omission of glutamine, which may mask any alterations, did not change this result. In addition to OCR, the extracellular acidification rate (ECAR) was also measured as an indicator of glycolysis within the cells and did not show any significant differences between the VARS2 KO^+/−^ and control cells (Figure S3B). This suggests that respiration in VARS2-deficient cells is still fully compensated and that mechanisms other than mitochondrial respiration are involved in adaptive changes in these cells.

### 2.5. VARS2 Depletion Leads to Activation of the Integrated Stress Response (ISR)

**Figure 4:**
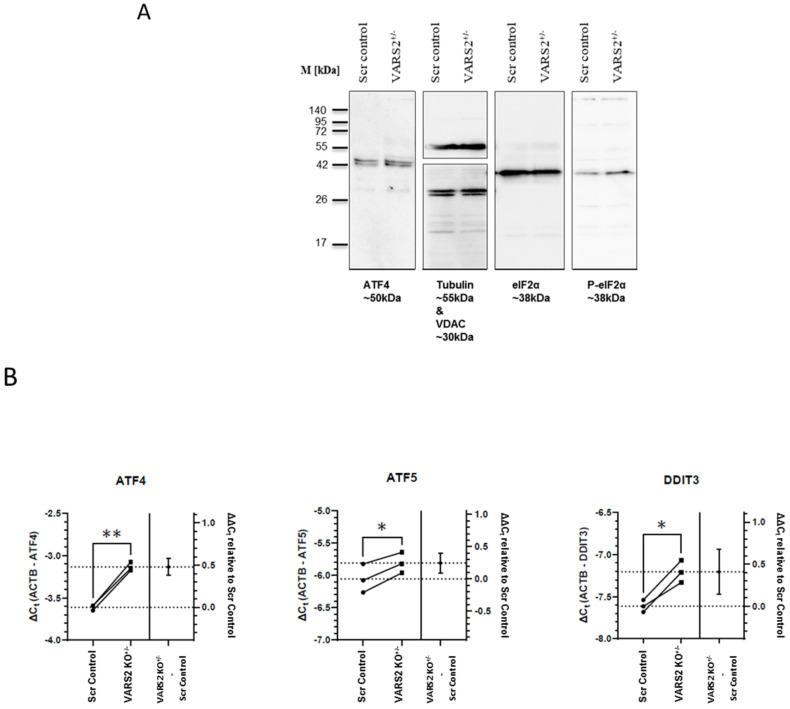
Western blot analyses showing a trend of a higher degree of eIF-2α phosphorylation in VARS2 KO^+/−^ cells (2.25x) compared to the controls (**A**). RT-qPCR results indicating a higher level of ATF4, ATF5 and DDIT3 (CHOP) transcripts in the VARS2^+/−^ compared to the control cell line (**B**). * = *p* < 0.05 and ** = *p* < 0.01.

To identify the molecular mechanism involved in maintaining homeostasis in VARS2-depleted cells, we assessed the activation of the integrated stress response (ISR), which can be triggered due to stresses including amino acid depravation. The activation of the ISR results in the phosphorylation of eukaryotic translation initiation factor 2 alpha (eIF2α) and leads to a decrease in global protein synthesis and the preferential translation of a subset of stress-response transcripts, including the activating transcription factor 4 (ATF4), that together promote cellular recovery [15]. Western blot analyses showed increased eIF-2α phosphorylation in VARS2 KO^+/−^ cells compared to control cells (2.25x) (Figure 4A). Using RT-qPCR, we found significant increases in the transcript levels of ATF4, as well as the ATF4 targets ATF5 and DDIT3 (CHOP), in VARS2 KO^+/−^ compared to the control cell line. The ATF4 transcript level was increased by ~39%, ATF5 by ~19%, and DDIT3 by ~33% (Figure 4B). ATF4 protein expression was also slightly higher in VARS2 KO^+/−^ compared to control cells. 

### 2.6. Disruptions in Mitochondrial FAO Are a Possible Pathomechanism Involved in Adaptive Changes in VARS2-Deficient Cells

**Figure 5:**
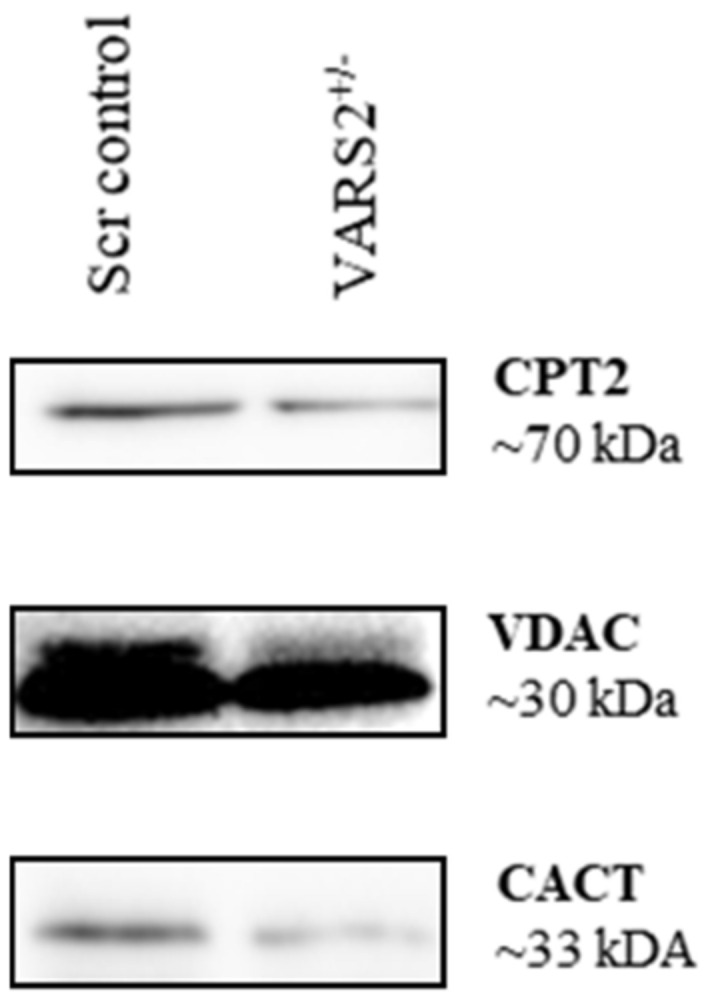
Western blot analyses revealing a trend of slightly lower protein levels of carnitine palmitoyltransferase 2 (CPT2) (64%) and carnitine/acylcarnitine translocase (CACT) (52%) in VARS2-depleted cells.

Western blot analyses revealed lower protein levels of carnitine palmitoyltransferase 2 (CPT2) (64%) and carnitine/acylcarnitine translocase (CACT) (0.52%) in VARS2 depleted cells, suggesting alterations in mitochondrial fatty acid oxidation (FAO) compared to the control cells (Figure 5).

## 3. Discussion

Defects in the mitochondria, the organelles responsible for energy production via the oxidative phosphorylation system (OXPHOS), appear to be the most common cause of adult and childhood neurometabolic diseases [16]. Such rare disorders are characterized by heterogeneous clinical presentations including hypotonia, developmental delay, lactic acidosis, failure to thrive, encephalopathy, and cardiomyopathy [6,13]. Mitochondrial diseases can be caused by mutations in both mitochondrial and nuclear DNA. Mitochondrial DNA (mtDNA) encodes 2 mitochondrial ribosomal and 22 transfer RNAs, as well as 13 of the 85 structural proteins of the respiratory chain (RC). The remaining RC proteins and over 250 proteins involved in the optimal maintenance and expression of the mitochondrial genome are encoded by nuclear genes and transported into mitochondria after cytosolic translation [10,17]. These include mitochondrial ribosomal proteins; initiation, elongation, and termination factors; tRNA-modifying enzymes; and aminoacyl-tRNA synthetases (mtARSs) [7,9,18]. The 20 mtARSs catalyze the attachment of each specific amino acid to its cognate tRNA via a two-step reaction. First, ATP reacts with the amino acid to build an aminoacyl adenylate. Second, the amino acid is ligated to the 3′-end of tRNA, forming the aminoacyl tRNA [19]. The mtARSs are also capable of correcting possible misloading with the wrong amino acid through their hydrolytic editing domains [20]. Mutations in mtARSs have been reported to cause Perrault, MLASA, and HUPRA syndromes, as well as encephalopathies, leukodystrophies, and cardiomyopathies [21].

Mutations in the VARS2 gene, located on chromosome 6p21.3, have been so far reported in 19 families, with more than 23 affected individuals [6,7,8,9,10,11,12,13,14,16,22,23]. Whereas homozygous carriers of c.1100C > T (p.Thr367Ile), the most common reported VARS2 variant, experienced fewer effects on the heart, hypertrophic cardiomyopathy was very often observed in compound heterozygotes carrying another variant alongside c.1100C > T or two other variants [6,8,9,12,13,14,16,22,23]. In a genome-wide association study (GWAS), we found an association between rs9262636, located in a non-coding region of chromosome 6, and dilated cardiomyopathy (DCM); through further eQTL analyses, we revealed a significant increase in the VARS2 mRNA levels in individuals carrying the minor allele G [1]. Thus, we postulated that VARS2 alterations may contribute to non-ischemic cardiomyopathies or influence patients’ clinical courses and outcomes, and we aimed to investigate the possible disease mechanisms. In this study, we found that zebrafish embryos lacking normal levels of VARS2 developed heart failure, cerebral edema, and curved backs, suggesting CNS involvement and skeletal muscle affection. We also showed the reduced enzymatic activity (reduced acylation/deacylation ratio) of valyl-tRNA in VARS2-depleted HEK293A cells. Moreover, we successfully generated the first in vitro VARS2-knockout cell model using CRISPR/Cas9 technology and characterized the heterozygous knockout by means of genomic PCR and sequencing, RT-qPCR, and western blot analysis. In these VARS2-depleted cells, we found a rearrangement of the electron transport chain (ETC) complexes in favor of complex III dimers (III2), III2 + IV complexes, and supercomplexes. Changes in five mitochondrial ETC complex activities have been reported in muscle homogenates of patients carrying VARS2 mutations. However, these changes were less uniform. Whereas Diodato et al. reported complex I deficiency with only 25% residual activity in an 8-year-old male patient with a homozygous missense mutation (c.1100C > T, p.Thr367Ile) in VARS2 [7], Pereira et al. reported normal levels of ETC complexes in their patients with combined oxidative phosphorylation deficiency 20 (COXPD20) who carried the same homozygous VARS2 mutation and died at 28 months [10]. Another 5-year-old patient with the same homozygous VARS2 mutation was reported to present a partial reduction in ETC complexes I  +  III in her muscle biopsy [11]. Taylor et al. reported the reduced activity of complexes I + IV in a male patient with a compound heterozygous mutation (c.1135G > A: p.Ala379Thr and c.1877C > A: p.Ala626Asp) [16]. This heterogeneity is a challenge to using abundance and activity measurements of ETC complexes as a diagnostic method. Our VARS2 KO^+/^^−^ cells showed no changes in subunit levels of each ETC complex but rather a rearrangement towards supercomplexes. It has been hypothesized that the organization of mitochondrial complexes as supercomplexes may offer structural or functional advantages, for instance preventing complex destabilization and degradation, enhancing electron transport efficiency and substrate channeling, or decreasing electron or proton leakages [24,25]. Seahorse analyses in the VARS2 KO^+/^^−^ cells revealed no significant abnormalities in the oxygen consumption rate (OCR) or extracellular acidification rate (ECAR), further supporting the notion that mechanisms other than mitochondrial respiration are involved in adaptive changes in VARS2-deficient cells.

Here, we showed that the depletion of VARS2, a mitochondrial aminoacyl-tRNA synthetase, resulted in the activation of the integrated stress response (ISR), which culminated in increased levels of eIF-2α phosphorylation and increased transcript levels of ATF4, ATF5, and DDIT3 (CHOP) in VARS2 KO^+/−^ cells. The phosphorylation of eIF-2α plays a central role in the ISR and leads to global translation attenuation in most proteins, with a few exceptions, such as activating transcription factor 4 (ATF4), which is actually preferentially translated [15]. It has been suggested that the activation of the ISR may be dependent on the degree of mitochondrial translation inhibition, indicating that mitochondrial translational machinery dysfunction promotes the homeostatic activation of the ISR [26].

Moreover, we demonstrated adaptive responses in mitochondrial fatty acid oxidation (FAO) in the VARS2 KO^+/−^ cells by showing their lower protein levels of carnitine palmitoyltransferase 2 (CPT2) and carnitine/acylcarnitine translocase (CACT) compared to the controls. Thus far, defects in FAO have not been investigated in patients carrying VARS2 mutations. However, fatty acid β oxidation has been shown to be the preferred energy-producing pathway in the mammalian heart, and it is essential for efficient cardiac pumping. Thus, inherited or acquired defects in mitochondrial FAO may cause hypertrophic (HCM) or dilated cardiomyopathies (DCM) or cardiac arrhythmias [27,28,29,30]. Treatments have been suggested for patients with CPT2 deficiency, including the avoidance of fasting and/or exercise, a low-fat diet enriched with medium-chain triglycerides, and carnitine supplementation [31]. Furthermore, such situations might be treated with targeted drugs that enhance glucose use and pyruvate oxidation energy at the expense of fatty acid oxidation and prevent the accumulation of long-chain acylcarnitines, which may result in increased cardiac conduction defects and arrhythmias [29].

## 4. Materials and Methods

### 4.1. Zebrafish Strains

Care and breeding of zebrafish, *Danio rerio*, were carried out as previously described [32]. This study was performed after obtaining institutional approvals that conformed to the Guide for the Care and Use of Laboratory Animals published by The US National Institute of Health (NIH Publication No. 85-23, revised 1996). For all Morpholino injection procedures, the TüAB wild-type strain, Heidelberg, Germany was used.

### 4.2. Morpholino Injection Procedures, Phenotyping, and RNA in Situ Hybridization

Morpholino-modified antisense oligonucleotides (Gene Tools) were directed against the splice-acceptor site of exon 3 (VARS2-MO (5′-TCA CGT CCT GTA AAA AGT TCA GGT T-3′)) of zebrafish vars2 (zvars2). The VARS2-MO and a standard control oligonucleotide (5′-CCT CTT ACC TCA GTT ACA ATT TAT A-3′) were diluted in 0.2 mol/liter KCl and microinjected into one-cell-stage zebrafish embryos. The microinjection was performed using a Femtojet Microinjection device (Eppendorf, Heidelberg). The capillary pressure was 15 hPa, and the injection time was 0.1s. The injection pressure was adjusted to the size of the needle. For sequencing zvars2, forward primer 5′-CCC GGA CAC AAG CAG AAA AAG CC-3′ and reverse primer 5′-TGC TCG GGA CTG AAG AAT TCC TGT -3′ were used. To measure the heart rate and ejection fraction, a 10 s video was recorded using a LEICA DM IRB microscope and LEICA DFC360 FX camera (Heidelberg). The heart rate was determined by counting the heart beats per minute. Fractional shortening was defined as $\mathrm{FS}=\frac{\mathrm{EDD}-\mathrm{ESD}}{\mathrm{EDD}}\times 100\%$, where FS is fractional shortening, LVEDD is left-ventricular end-diastolic diameter (mm), and LVESD is left-ventricular end-systolic diameter (mm). The diameters were measured with the help of zebraFS software (http://www.benegfx.de). Whole-mount in situ hybridization of zebrafish embryos was performed as previously described on embryos fixed in 4% paraformaldehyde [33].

### 4.3. Manipulating the Expression of VARS2 Gene in HEK293A Cells

Human embryonic kidney cells (HEK293A) were purchased from ThermoFisher scientific (#R70507, Heidelberg, Germany). Cells were cultured in DMEM (Gibco, #21969-035) supplemented with 10% fetal calf serum (FCS) (c.c.pro, #S-10-L), 1% penicillin and streptomycin (Gibco, #15070-063), and L-glutamine (Gibco, #20530-024). Cultivation was performed at 37 °C and 5% CO2 in an incubator. The cells were distributed at approx. 80% confluence in a ratio of 1:10 among new cell-culture flasks. For detachment, the cells were incubated with 0.25% trypsin for 3 min at 37 °C and then collected, centrifuged (5 min, 1000 g), and distributed among new cell culture flasks. Initially, three different siRNAs complementary to the mRNA of VARS2 and one siRNA without complementary RNA (so-called scrambled siRNA, siSCR) were purchased from Sigma-Aldrich, Taufkirchen, Germany (from 5′→3′—siVARS2-1: GUC AAC UGG UCA UGU GCU U[dT][dT]; siVARS2-2: CGC UUU AUC CUC AAU GCU U[dT][dT]; siVARS2-3: CUA AGG AGU UAG UAU UGU A[dT][dT]; and siSCR: UCU CUC ACA ACG GGC AUU U[dT][dT]). The transfection was carried out according to the Lipofectamine RNAiMax protocol (Invitrogen, Waltham, MA, USA, #13778). The efficiency of the knockdown was determined 24 h later. We excluded siVARS2-3 from further analysis because of severe cell toxicity.

### 4.4. Immunohistochemical Studies in HEK293A Cells

To first confirm the subcellular localization of VARS2 in HEK293A cells, they were examined immunocytochemically using an anti-VARS2 antibody (Proteintech #15776-1-AP, Polyclonal, Germany). Mitochondria were stained using MitoTracker Deep Red FM (ThermoFisher #M22426, Germany), and nuclei were stained using DAPI. β-actin, a structural protein of the cytoskeleton, served as a control (Sigma-Aldrich #A5441). VARS2 antibodies were detected using a fluorescein-labelled secondary antibody (anti-mouse FITC, Sigma Aldrich #F5262, Germany).

### 4.5. Primers

The following RT-qPCR primers were used to assess transcript levels in HEK293A cells. VARS2 for siRNA experiments: forward 5′-CAG CAT CTC GGT TGC CCC-3′ and reverse 5′-CTT TGG TGA GCT GGT ACG GT-3′; VARS2 for CRISPR/Cas9 experiments: forward 5′-GAG GTA GCA GCG GAA CTG AC-3′ and reverse 5′- GTG ACA GGG GGT AGA AAC GA-3′ (VARS2 FR4), forward 5′-TGT GTA TCC CAC CTC CCA AT-3′and reverse 5′- TCC ACC ACA GCT TGT GTA GC-3′ (VARS2 FR5), and forward 5′- CTA CGA AAC CCG GTG AAA AG-3′ and reverse 5′-TGA AGA AGC CCT CTC GTA CC-3′ (VARS2 FR8); ATF4: forward 5′-CAG CAA GGA GGA TGC CTT CT-3′ and reverse 5′-CCA ACA GGG CAT CCA AGT C-3′; ATF5: forward 5′-GAG CCC CTG GCA GGT GAT-3′ and reverse 5′-CAG AGG GAG GAG AGC TGT GAA-3′; DDIT3: forward 5′-GCA AGA GGT CCT GTC TTC AGA TG-3′ and reverse 5′-CTC AGT CAG CCA AGC CAG AGA-3′; ACTB: forward 5′-AGA GCT ACG AGC TGC CTG AC-3′ and reverse 5′-AGC ACT GTG TTG GCG TAC AG-3′.

The following primers were used in genomic PCRs to genotype the gene-edited VARS2 allele in HEK293A cells: Forward 5′-AAG GTT AGG GGT CAG ACA GC-3′ and reverse 5′-CCG TAG CTC CAA TCC TTC CA-3′, forward 5′-CAA CCT CCC CTT CTA CGA GC-3′ and reverse 5′-GCG AGT GGA AGA AGG TGA GA-3′, and forward 5′-AAG GTT AGG GGT CAG ACA GC-3′ and reverse 5′-GCG AGT GGA AGA AGG TGA GA-3′.

### 4.6. Northern Blot

For a detailed description of the northern blot protocol, please refer to the Supplementary Materials.

### 4.7. Establishing a Monoclonal VARS2-Knockout HEK293A Cell Line

The VARS2^+/^^−^ knockout was achieved with an “HDR-mediated CRISPR kit” from Rockville, MD, USA) following the manufacturer’s CRISPR/Cas9 genome-editing application guide with a few adaptations. For a detailed explanation, please refer to the Supplementary Materials.

### 4.8. Isolation of Mitochondria

The mitochondria were extracted via differential centrifugation after homogenization of the cells in THE buffer (300 mM trehalose, 10 mM KCl, 10 mM HEPES, 1 mM EDTA, 1 mM EGTA, 0.5 mM PMSF, pH 7.4; 1× complete protease inhibitor from Roche, 1× phosphatase inhibitor from Roche, and mechanical disruption using a Dounce homogenizer). All centrifugation steps were performed at 4 °C, and we included a clarifying step at 400 g for 5 min, followed by a 800 g centrifugation step for 5 min and a centrifugation step at 18,620 g for 10 min. The supernatant resulting from this step corresponded to the cytosolic cell fraction, and the pellet corresponded to the mitochondrial fraction. The pellet was then washed in THE buffer and subjected to a final centrifugation step at 18,620 g for 5 min. The resulting pellet, consisting of the isolated mitochondria, was resuspended in THE buffer. For experiments run on BN-PAGE, 30 µg mitochondria or in one case 19 µg was run per lane. All SDS-PAGE processes performed with the mitochondrial cell fraction included 20 µg or 15 µg mitochondria per lane. For SDS-PAGE processes with whole-cell lysate, around 60 µg or 75 µg whole-cell lysate was run per lane. For quantification of VARS2 and VDAC protein levels, 10 µg whole-cell lysate was run on each lane.

### 4.9. Blue Native PAGE (BN-PAGE)

Mitochondrial membranes were solubilized in either a 1% digitonin solubilization buffer (20 mM Tris HCl pH 7.4, 0.1 mM EDTA, 50 mM NaCl, 10% glycerol, and 1 mM PMSF) for at least 30 min at 4 °C, and the non-solubilized material was removed by a clarifying spin at 20,000 g for 10 min at 4 °C. 10× loading dye (5% Coomassie blue G-250, 500 mM ε-amino n-caproic acid, 100 mM Bis-Tris pH 7.0) was added to the supernatant before separation on a 4–13% polyacrylamide gradient gel at 4 °C. After running the BN-PAGE for 1 h using a BN anode buffer (50 mM Bis-Tris HCl pH 7.0) and a BN cathode buffer (50 mM Tricaine pH 7.0, 150 mM Bis-Tris, 0.02% Coomassie blue G-250), the BN cathode buffer was replaced by a new BN cathode buffer without the Coomassie blue G-250 [34]. Upon completion of the electrophoresis, the remaining steps were identical to western blotting.

### 4.10. Seahorse

The oxygen consumption rate (OCR) and extracellular acidification rate (ECAR) were measured with a Seahorse XFe96 Analyzer from Agilent. In brief, 15,000 cells per well were seeded in an Agilent Seahorse 96-well XF Cell Culture Microplate in standard 4.5 g/L glucose DMEM. On the day the assay was run, the medium was exchanged for Seahorse XF base medium supplemented with 10 mM (1.8 g/L) glucose, 1 mM pyruvate, and 2 mM glutamine, unless otherwise specified. The plate was then placed in an incubator at 37 °C without CO_2_ for 1 h before running the assay. Three OCR and ECAR measurements were performed per metabolic state, beginning with the basal measurements. The different metabolic states were induced by the subsequent addition of 3 µM oligomycin, 2 µM FCCP, 1 µM antimycin A, and 2 µM rotenone. The assay results were analyzed using Wave 2.6.1 desktop software and exported to an Excel spreadsheet for graphical presentation.

### 4.11. Statistical Analyses

Statistical differences were determined using the Student’s *t*-test or a two-way ANOVA, where *p* < 0.05 was considered statistically significant. Multiple testing was corrected using the Benjamini and Hochberg method. For a detailed description of the statistical analyses used for RT-qPCR and western blot data, please refer to Supplementary Materials.

## 5. Conclusions

The activation of the integrated stress response (ISR) and alterations to mitochondrial fatty acid oxidation (FAO) are two possible mechanisms involved in the adaptive changes in VARS2-depleted cells. Considering these results and based on our previous findings, we hypothesize that VARS2 alterations may contribute to non-ischemic cardiomyopathy or influence patients’ clinical courses and outcomes; therefore, we suggest further investigation.

## Data Availability

Not applicable.

## Supplementary Materials

The following supporting information can be downloaded at: https://www.mdpi.com/article/10.3390/ijms23137327/s1 [35,36,37].
