# Supplementary material for: VARS2 Depletion Leads to Activation of the Integrated Stress Response and Disruptions in Mitochondrial Fatty Acid Oxidation
Source: Int J Mol Sci. 2022 Jun 30;23(13):7327. doi: 10.3390/ijms23137327 (PMC9267100; doi:10.3390/ijms23137327)
Supplement: Supplementary file 1 [file ijms-23-07327-s001.zip › ijms-1789489-SI.pdf]

Northern blot analysis

tRNA isolation

First, cells were washed twice with 37 °C 300 mM sodium acetate pH 4.5 and then detached with a cell scraper and transferred to 500 µl 300 mM sodium acetate pH 4.5 in a microreaction vessel. Three wells were collected in a microreaction vessel. The cells were centrifuged at 5000 rpm for 5 min at 4 °C, the supernatant discarded, the remaining cells resuspended in 300 µl 300 mM sodium acetate pH 4.5 with 10 mM EDTA and 300 µl phenol: chloroform (pH 4.9) (Invitrogen #AM9720) added. To disrupt the cells, they were shaken well five times for 30 seconds and the cells were kept on ice between steps. To separate the phases, centrifugation was performed for 15 min at 14000 rpm and 4 °C and the upper phase with the RNA was transferred to a new microreaction tube. After the addition of 300 µl phenol: chloroform (pH 4.9), centrifugation was repeated for 15 min at 14000 rpm and 4 °C and the upper phase was transferred to a new microreaction tube. For precipitation of the RNA, three times the volume of pure ethanol was added, mixed briefly and centrifuged for 25 min at 14000 rpm and 4 °C. The RNA was precipitated a total of two times. Precipitation was performed twice in total, i.e. the pellet was dissolved again in 60 µl 300 mM sodium acetate pH 4.5 and 300 µl 100% ethanol after the first precipitation. For deacylation, the tRNA was incubated in Tris/HCl with pH 8 at 37 °C for 2h after the first precipitation and precipitated again with ethanol. Finally, the precipitated RNA was dissolved in 10 mM sodium acetate pH 4.5 and the concentration was determined on a spectrometer.

RNA electrophoresis

First, the gel was poured and allowed to set overnight at 4°C. The RNA was brought to a volume of 5 µl with 10 mM sodium acetate pH 4.5 and mixed in a ratio of 1:1 with 2X loading buffer. The RNA was then loaded onto the gel. The buffer used for electrophoresis was 0.3 M sodium acetate at pH 5.0.

Denaturing gel:

- 3 ml 1M sodium acetate pH5.0

- 4.2g urea

- 3.5 ml 40% polyacrylamide (BioRad #1610148)

- Make up to 10 ml with DEPC water and adjust pH to 5.0.

Native gel:

- 3 ml 1M sodium acetate pH5.0;

- 3.5 ml 40% polyacrylamide (BioRad #1610148);

- make up to 10 ml with DEPC water and adjust pH to 5.0.

At the beginning, electrophoresis was started with 20 V and the voltage was increased to 40 V in intervals of one hour and steps of 5-10 V. The electrophoresis was continued for about 24 hours. In total, the RNA was run for about 24 h at 4°C until the xylene cyanol reached the end of the gel. Since the pH of the buffer in the inner chamber increases during electrophoresis, it was stopped briefly every 8 h and the buffer was redistributed to the chambers.

Preparation of DIG labelled probes

For the preparation of the DIG labelled RNA probes the DIG RNA labelling kit from Roche (#11175025910) was used. A purified PCR product was used as substrate. The reverse primer of this PCR carries the sequence of the T7 promoter at the 5'end, so this was inserted into the product during PCR. Following primers were used: from 5‘→3‘: Val- tRNA fw: GCT TAA CAC AAA GCA CCC AAC and Val- tRNA rv: GGA TCC TAA TAC GAC TCA CTA TAG GCA AGT TAA GTT GAA.

In vitro transcription reaction set-up:

- 2 µl 10X dNTP labelling mixture
- 2 µl 10X transcription buffer
- 2 µl T7 RNA polymerase
- 1 µl RNase inhibitor
- 1 µg PCR product
- Make up to 20 µl with H20

The reaction was incubated for 2 h at 37 °C. Then incubated for 15 min with 2 µL DNase I at 37 °C and stopped with 2 µL 0.2 M EDTA. The product was diluted 1:10 in DEPC treated water and stored at -80 °C.

RNA blotting and hybridisation

The transfer of the RNA on a nylon membrane was done with the Power Blotter Station from Thermofischer (15V for a 1h with transfer buffer). To fix the RNA on the membrane, UV crosslinking with UV Stratalinker 1800 (Stratagene) was performed. The membrane was put into the hybridisation buffer for 2 h at 42 °C. Subsequently, 2.5 µl of 1:10 diluted probe was added to 3 ml of hybridisation buffer and incubated at 42 °C overnight. For detection, the DIG wash and blocking buffer set from Roche (#11585762001) and the DIG luminescent detection kit (#11363514910) were used, whereby the membrane was first briefly given wash buffer for 5 min. Then the membrane was blocked with 100 ml blocking reagent for 1 h and incubated with a DIG specific antibody (diluted 1:10000 in 20 ml blocking reagent) for 1 h. Then the membrane was incubated twice for 15 min with wash buffer. This was followed by two 15 min incubations with wash buffer. CSPD from Roche was used for the detection of the DIG labelled RNA probe. The membrane was placed in a cassette with an appropriate film, which

was then developed. For removal of the probe, the membrane was placed twice in stripping buffer at 65 °C for 45 minutes.

Establishing a monoclonal VARS2 knockout HEK293A cell line

The *VARS2* knockout was achieved with an "HDR-mediated CRISPR kit" from OriGene Terchnologies Inc. (USA) following the manufacturer's "CRISPR/Cas9 Genome Editing Application Guide" for "HDR-mediated CRISPR knockout kits" with a few adaptations. The transfection reagent Turbofectin 8.0 was used for the co-transfection reactions. Puromycin selection with 0.5 µg/ml puromycin was applied 48 hours post co-transfection. Following puromycin selection, in a process called limiting dilution, the cells were serially diluted and seeded on 96-well plates to generate cell lines stemming from a single cell.

After expansion of monoclonal cell lines, DNA was extracted using the "QIAamp DNA Blood Mini" kit from Qiagen (Cat. no. 51106) following the protocol for cultured cells described in the manufacturer's handbook. Genomic PCRs were performed to screen for cell lines containing a VARS2 knockout using a PrimeSTAR GXL DNA Polymerase from Takara (Cat. #R050A) following the "Rapid PCR Protocol" explained in the product manual.

For interesting cell lines, the knockout was verified on a protein and an RNA level via Western blotting and RT-qPCR respectively. Protein was extracted from whole cell lysate using a RIPA lysis buffer (150 mM NaCl, 1% TritonX-100, 0.5% Sodium deoxycholate, 0.1% SDS, 50mM Tris HCl, 1x phosphatase inhibitor (PhosSTOP™ ,Ref. 04 906 837 001 Sigma), 1x protease inhibitor cocktail (cOmplete™ Protease Inhibitor Cocktail, Ref. 04 693 116 001 Sigma). The lysate was centrifuged at 20 000 g for 10 min at 4°C and the supernatant transferred into a new tube and measured using an RC DC™ protein assay (RC DC™ Protein Assay Kit, Cat. No. 5000121) following the manufacturer's instructions for the "Microfuge

Tube Assay Protocol”. Western blotting was performed following the lab’s internal standard protocol.

Statistical analyses

Statistical analyses for the RT-qPCR data

For each target, prior to relative quantification of gene expression, an RT-qPCR with a 4-fold cDNA dilution series (end dilution in well 1:40, 1:400, 1:4 000 and 1:40 000) of a scrambled control cDNA sample was run to check for primer efficiency. The cDNA dilution factor was plotted against the average Ct value of the technical triplicate and a trend line inserted using Excel. The efficiency in % was calculated with the formula $(10^{1/m} - 1) * 100\%$ with m being the slope of the trend line [1]. Primer pairs with ~ 90-110 % efficiency and acceptable melting curves (no primer dimers) were selected for the relative quantification of gene expression.

Relative gene expression was determined using a slight adaptation of the $\Delta\Delta C_t$ method described by [2]. Using this method, the C_t values of each target are normalized to an endogenous reference (ΔC_t). The suitability of the reference was determined by comparing the average C_t values across the different sample groups and the reference gene with an acceptably minimal C_t difference was chosen. For illustration purposes, ΔC_t is calculated as $C_{t,\text{reference gene}} - C_{t,\text{ target}}$ like in [3] as opposed to $C_{t,\text{ target}} - C_{t,\text{reference gene}}$ as originally proposed by [2]. Thus, here, more positive ΔC_t values indicate higher levels of the target RNA.

For each RT-qPCR plate run for the purpose of relative quantification of gene expression, three RNA samples per cell line were extracted in parallel. Following the reverse transcriptase reaction, each sample was pipetted in technical triplicates. For analyses, the mean C_t was determined from the technical triplicates per sample and target, and used to calculate the ΔC_t value, resulting in three ΔCt values for each cell line and target per plate. The mean of these three ΔC_t values was used for statistical analyses. Three such plates were run (n=3) and a paired t-test with equal variances was then performed on the three mean ΔC_t values (one per plate) between the cell line of interest and the calibrator, i.e. the control cell

line. The ΔC_t value of the cell line of interest and the ΔC_t value of the control cell line stemming from the same plate were paired, resulting in three corresponding $\Delta\Delta C_t$ values ($\Delta C_{t,\ \text{cell line of interest}} - \Delta C_{t,\ \text{calibrator}}$).

Assuming an ideal primer efficiency of 100 %, the formula $2^{\Delta\Delta Ct}$ was used to obtain the fold change. Statistics were performed on the normally distributed ΔC_t values as opposed to the fold change values which aren't normally distributed following transformation with "2^".

Western blot quantification and statistical analyses

For relative quantification of protein levels, densitometry analyses were performed using ImageJ for the blots developed in Heidelberg or using the Image Lab software by Bio-Rad for the blots developed in Würzburg. Data was normalized with a loading control. In brief, the ratio of the target protein stemming from a sample of interest to the target protein stemming from the reference sample was calculated and then normalized by the ratio calculated for the loading control between the two samples.

Furthermore, each blot had multiple images corresponding to different film exposure times. Based on the protein band to be measured, an image was selected so the bands of interest were neither under- or oversaturated. As a side note this meant that on occasion the housekeeping protein was quantified on an image with a different exposure time than the target protein.

For each Western blot gel run in Heidelberg, three protein samples per cell line were extracted in parallel. Thus, in step 1 and 2 of "Data Analysis with loading-control bands" of the protocol, the mean percent value across the three lanes loaded with the control cell line

were used as the standard to be divided by. After step 3, the mean out of the three values per cell line was formed, resulting in one value per cell line per Western blot.

Table S1

Table Analyzed	basal OCR				
Two-way ANOVA	Ordinary				
Alpha	0,05				
Source of Variation	% of total variation	P value	P value summary	Significant?	
Interaction	0,02231	0,9956	ns	No	
Row Factor	5,891	0,3255	ns	No	
Column Factor	2,544	0,3239	ns	No	
ANOVA table	SS (Type III)	DF	MS	F (DFn, DFd)	P value
Interaction	6,689	2	3,344	F (2, 36) = 0,004387	P=0,9956
Row Factor	1766	2	883	F (2, 36) = 1,158	P=0,3255
Column Factor	762,7	1	762,7	F (1, 36) = 1	P=0,3239
Residual	27445	36	762,4		
Number of missing values	6				
Table Analyzed	maximal OCR				
Two-way ANOVA	Ordinary				
Alpha	0,05				
Source of Variation	% of total variation	P value	P value summary	Significant?	
Interaction	1,395	0,7445	ns	No	
Row Factor	4,857	0,3653	ns	No	
Column Factor	9,347	0,0535	ns	No	
ANOVA table	SS (Type III)	DF	MS	F (DFn, DFd)	P value
Interaction	244,5	2	122,2	F (2, 36) = 0,2974	P=0,7445
Row Factor	851,4	2	425,7	F (2, 36) = 1,036	P=0,3653
Column Factor	1638	1	1638	F (1, 36) = 3,987	P=0,0535
Residual	14795	36	411		
Number of missing values	6				

References

1. Rutledge, R.G.; Côté, C. Mathematics of quantitative kinetic pcr and the application of standard curves. *Nucleic Acids Research* **2003**, *31*, e93-e93.
2. Livak, K.J.; Schmittgen, T.D. Analysis of relative gene expression data using real-time quantitative pcr and the 2−δδct method. *Methods* **2001**, *25*, 402-408.
3. Biasin, V.; Wygrecka, M.; Marsh, L.M.; Becker-Pauly, C.; Brcic, L.; Ghanim, B.; Klepetko, W.; Olschewski, A.; Kwapiszewska, G. Meprin β contributes to collagen deposition in lung fibrosis. *Scientific Reports* **2017**, *7*, 39969.

Figure S1

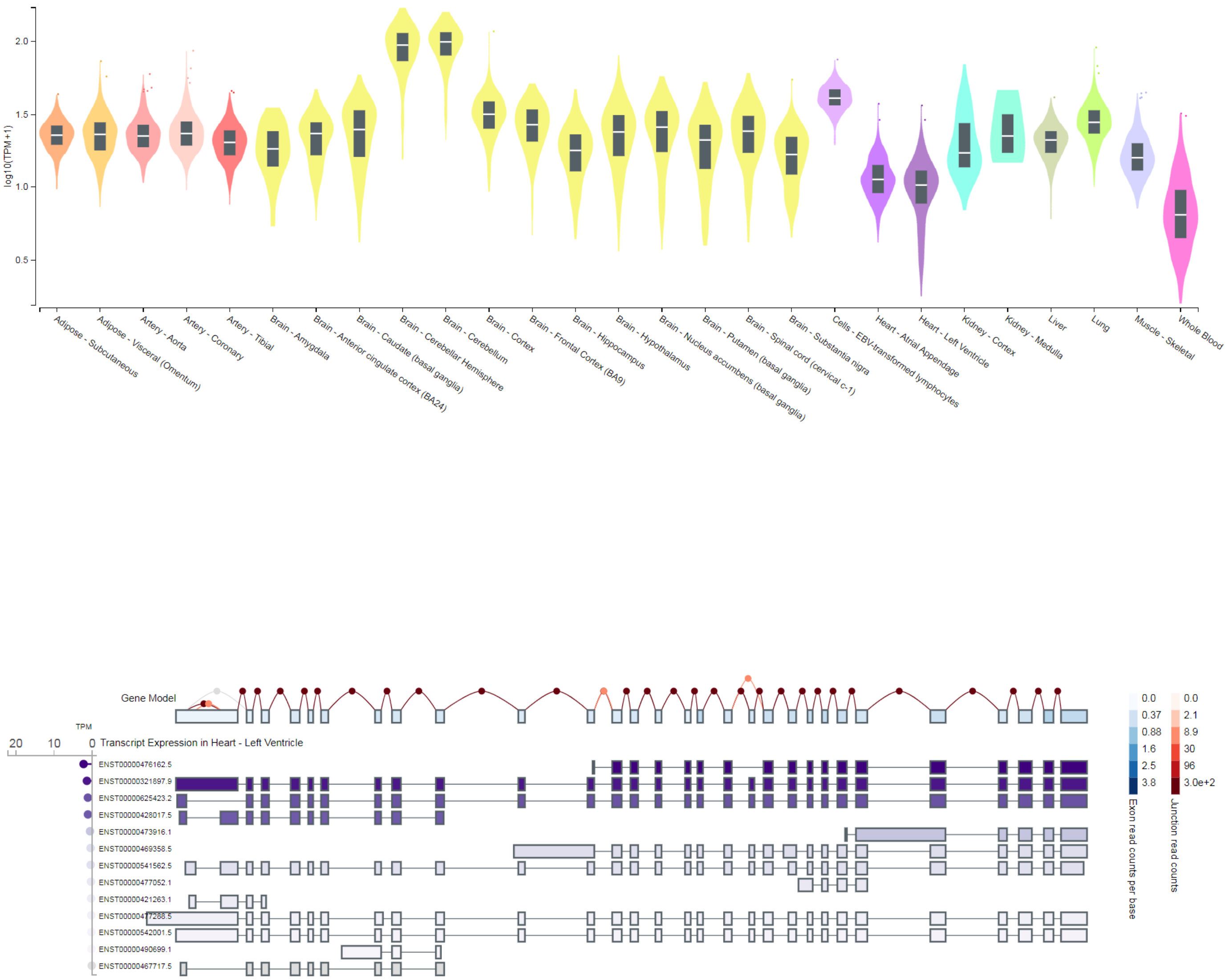

log10(TPM+1)
2.0
1.5
1.0
0.5
Adipose - Subcutaneous
Adipose - Visceral (Omentum)
Artery - Aorta
Artery - Coronary
Artery - Tibial
Brain - Amygdala
Brain - Anterior cingulate cortex (BA24)
Brain - Caudate (basal ganglia)
Brain - Cerebellar Hemisphere
Brain - Cerebellum
Brain - Cortex
Brain - Frontal Cortex (BA9)
Brain - Hippocampus
Brain - Hypothalamus
Brain - Nucleus accumbens (basal ganglia)
Brain - Putamen (basal ganglia)
Brain - Spinal cord (cervical c-1)
Brain - Substantia nigra
Cells - EBV-transformed lymphocytes
Heart - Atrial Appendage
Heart - Left Ventricle
Kidney - Cortex
Kidney - Medulla
Liver
Lung
Muscle - Skeletal
Whole Blood
Gene Model
TPM
20
10
0
Transcript Expression in Heart - Left Ventricle
ENST00000476162.5
ENST00000321897.9
ENST00000625423.2
ENST00000428017.5
ENST00000473916.1
ENST00000469358.5
ENST00000541562.5
ENST00000477052.1
ENST00000421263.1
ENST00000477288.5
ENST00000542001.5
ENST00000490699.1
ENST00000467717.5
0.0
0.37
0.88
1.6
2.5
3.8
Exon read counts per base
0.0
2.1
8.9
30
96
3.0e+2
Junction read counts

Figure S2

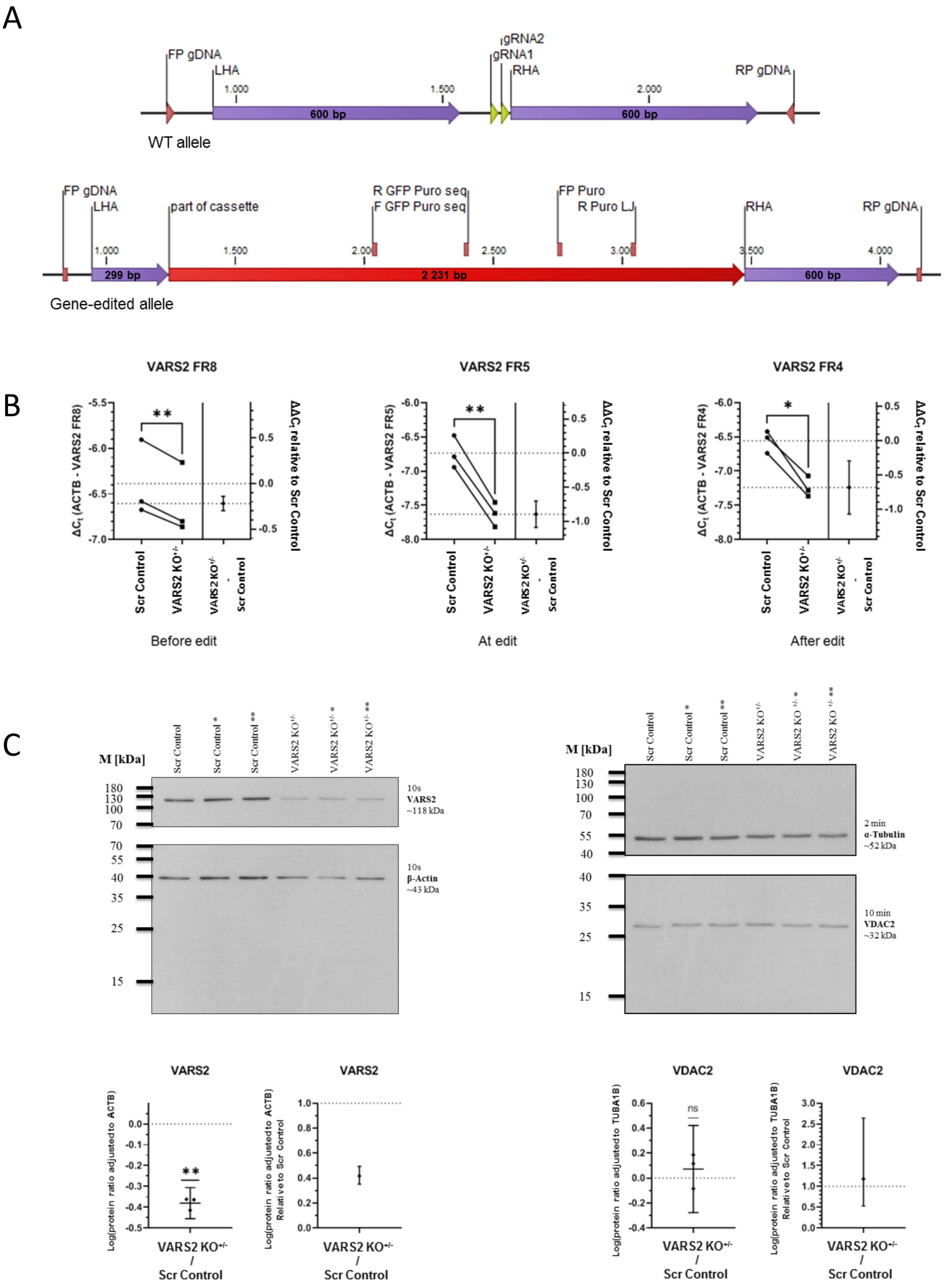
A
FP gDNA
LHA
gRNA2
gRNA1
RHA
RP gDNA
1.000
1.500
2.000
600 bp
600 bp
WT allele
FP gDNA
LHA
part of cassette
R GFP Puro seq
F GFP Puro seq
FP Puro
R Puro LJ
RHA
RP gDNA
1.000
1.500
2.000
2.500
3.000
3.500
4.000
299 bp
2 231 bp
600 bp
Gene-edited allele
B
VARS2 FR8
VARS2 FR5
VARS2 FR4
ΔCt (ACTB - VARS2 FR8)
ΔCt (ACTB - VARS2 FR5)
ΔCt (ACTB - VARS2 FR4)
ΔΔCt relative to Scr Control
Scr Control
VARS2 KO+/-
VARS2 KO+/- - Scr Control
Before edit
At edit
After edit
C
M [kDa]
Scr Control
Scr Control *
Scr Control **
VARS2 KO+/-
VARS2 KO+/- *
VARS2 KO+/- **
10s
VARS2
~118 kDa
10s
β-Actin
~43 kDa
2 min
α-Tubulin
~52 kDa
10 min
VDAC2
~32 kDa
VARS2
VARS2
VDAC2
VDAC2
Log(protein ratio adjusted to ACTB)
Log(protein ratio adjusted to ACTB) Relative to Scr Control
Log(protein ratio adjusted to TUBA1B)
Log(protein ratio adjusted to TUBA1B) Relative to Scr Control
ns
VARS2 KO+/- / Scr Control

Figure S3

A

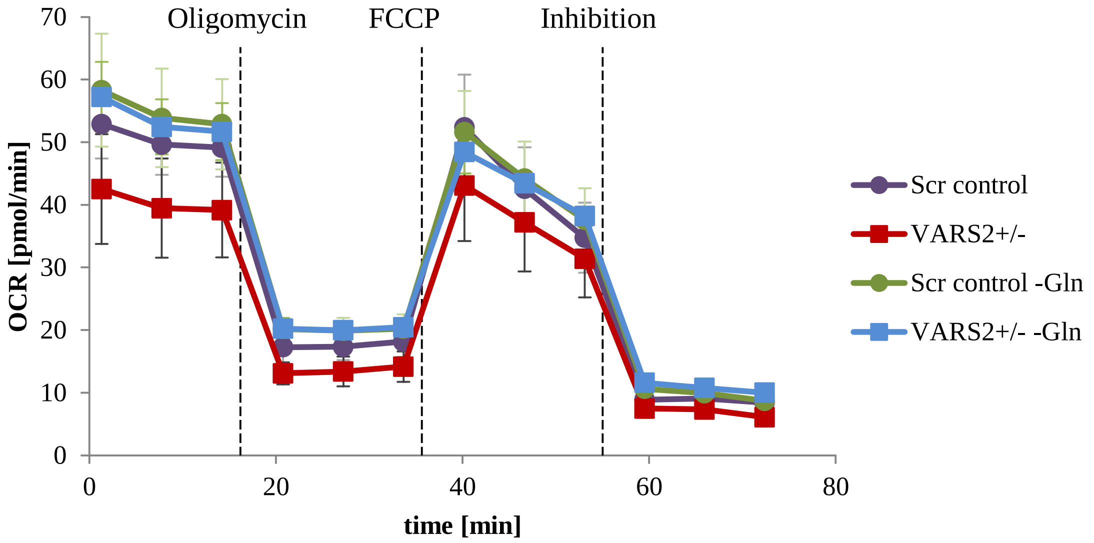
Oligomycin
FCCP
Inhibition
OCR [pmol/min]
time [min]
Scr control
VARS2+/-
Scr control -Gln
VARS2+/- -Gln

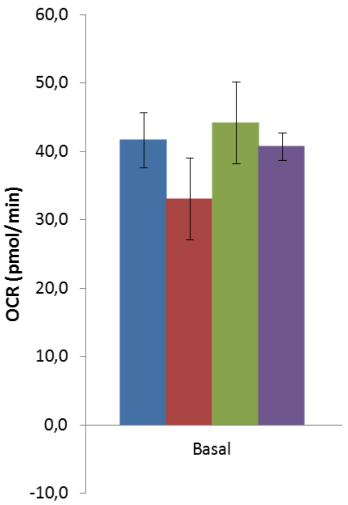
OCR (pmol/min)
Basal

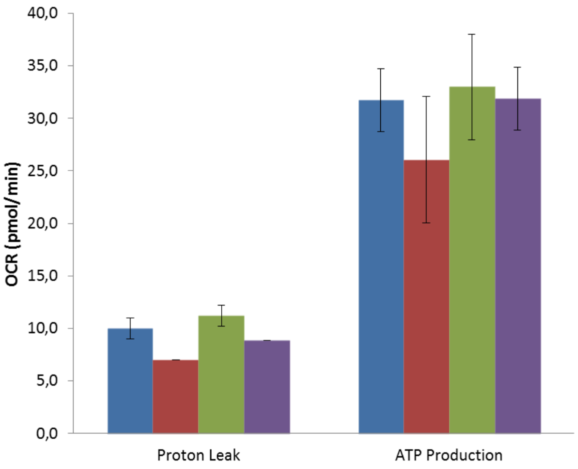
OCR (pmol/min)
Proton Leak
ATP Production

B

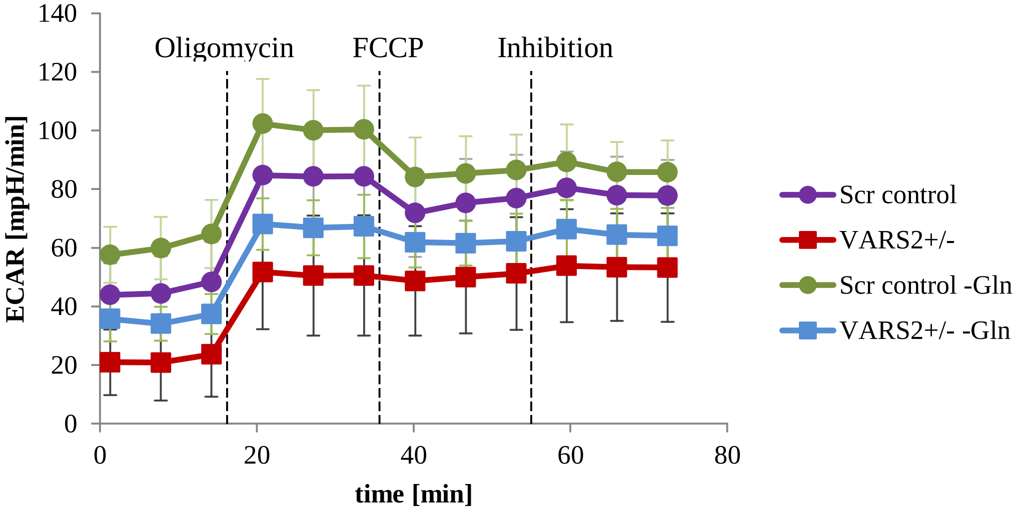
Oligomycin
FCCP
Inhibition
ECAR [mpH/min]
time [min]
Scr control
VARS2+/-
Scr control -Gln
VARS2+/- -Gln
